# Biochemical Evaluation of the Antioxidant Effects of Hydroxytyrosol on Pancreatitis-Associated Gut Injury

**DOI:** 10.3390/antiox9090781

**Published:** 2020-08-22

**Authors:** Roberta Fusco, Marika Cordaro, Rosalba Siracusa, Ramona D’Amico, Tiziana Genovese, Enrico Gugliandolo, Alessio Filippo Peritore, Rosalia Crupi, Daniela Impellizzeri, Salvatore Cuzzocrea, Rosanna Di Paola

**Affiliations:** 1 Department of Chemical, Biological, Pharmaceutical and Environmental Sciences, University of Messina, 98166 Messina, Italy; rfusco@unime.it (R.F.); rsiracusa@unime.it (R.S.); rdamico@unime.it (R.A.); tgenovese@unime.it (T.G.); egugliandolo@unime.it (E.G.); aperitore@unime.it (A.F.P.); dipaolar@unime.it (R.D.P.); 2 Department of Biomedical, Dental and Morphological and Functional Imaging University of Messina, Via Consolare Valeria, 98125 Messina, Italy; cordarom@unime.it; 3 Department of Veterinary Sciences, University of Messina, 98168 Messina, Italy; rcrupi@unime.it; 4 Department of Pharmacological and Physiological Science, Saint Louis University School of Medicine, Saint Louis, MO 63104, USA

**Keywords:** medicinal plants, hydroxytyrosol, antioxidant

## Abstract

Acute pancreatitis is a severe abdominal pathology often associated with several complications including gut dysfunction. Oxidative stress is one of the most important pathways involved in this pathology. Hydroxytyrosol (HT), a phenolic compound obtained from olive oil, has shown anti-inflammatory and antioxidant properties. We evaluated the effects of HT administration on pancreatic and intestinal injury induced by caerulein administration. CD1 female mice were administered caerulein (50 μg/kg) for 10 h. HT treatment (5 mg/kg) was performed 30 min after the first caerulein injection and for two consecutive hours afterwards. One hour after the last caerulein injection, mice were sacrificed and serum, colon and pancreatic tissue samples were collected. HT was able to reduce the serum hallmarks of pancreatitis (amylase and lipase), histological damage score in both pancreas and colon tissue, inflammatory cells recruitment (mast cells) in both injured tissues, intrapancreatic trypsin activity and overexpression of the adhesion molecules (Intercellular Adhesion Molecule-1 (ICAM-1) and P-selectin) in colon. Additionally, HT reduced cytokine (interleukin 1 beta (IL- 1β), interleukin 6 (IL-6) and tumor necrosis factor alpha (TNF-α)) levels in serum, pancreas and colon tissue and chemokine release (monocyte chemotactic protein-1 (MCP1/CCL2)) in pancreas and colon tissue. HT decreased lipid peroxidation and oxidative stress (superoxide dismutase (SOD), glutathione peroxidase (GPx), glutathione reductase (GR) and glutathione S-transferase (GST) activity) by enhancing the nuclear factor erythroid 2-related factor 2 (Nrf2) and heme oxygenase 1 (HO-1) in both injured tissues. Moreover, HT preserved intestinal barrier integrity, as shown by the diamine oxidase (DAO) serum levels and tight junction (zonula occludens (ZO) and occludin) expression in pancreas and colon. Our findings demonstrated that HT would be an important therapeutic tool against pancreatitis-induced injuries in the pancreas and gut.

## 1. Introduction

Acute pancreatitis is an inflammatory pathology characterized by elevation of serum pancreatic enzymes and acute abdominal pain [1]. Although the pancreas is primarily an exocrine gland, secreting a variety of digestive enzymes (trypsin, chymotrypsin and carboxypeptidase), it has an endocrine function. Its pancreatic islets secrete the hormones glucagon, insulin, somatostatin and pancreatic polypeptide. The normal secretion of digestive enzymes is modified during pancreatitis. Digestive enzymes, which are exported outside the cells and lysosomal hydrolases, which are transported into lysosomes, are normally separated from each other while they pass through the Golgi system [2,3]. In injured pancreas, the separation of digestive enzymes from lysosomal hydrolases is defective and, as a result, both types of enzyme become colocalized within intracellular vacuoles [2]. This colocalization phenomenon may result in premature activation of digestive enzymes. Subsequent rupture of these vacuoles liberates the activated digestive enzymes in the cytoplasmic space, followed by a cascade of events that finally results in acute pancreatitis [4]. The activation of pancreatic enzymes in and around the pancreas and bloodstream results in coagulation necrosis of the pancreas and necrosis and hemorrhage of peripancreatic and peritoneal adipose tissue. Recently, pancreatitis rate has considerably increased [5]. Moreover, this disease may be self-limited or develop as a severe condition with up to 30% lethality [6]. Although its etiology remains elusive, accumulated evidence has linked acute pancreatitis to inflammation of acinar cells, infiltration by innate immune cells and derived inflammatory mediators [7,8]. Additionally, intestinal dysfunction and secondary inflammatory issues aggravate acute pancreatitis retroactively and are prodromes of systemic complications [9,10]. Acute pancreatitis is often related to multiple organ dysfunction syndrome (MODS) and systemic inflammatory response syndrome (SIRS) [11]. Several works describe the pancreatitis-associated failure of the gastrointestinal tract. This is characterized by the release of endotoxins from the intestinal lumen and the translocation of enteric bacteria to systemic circulation. These events increase the intestinal permeability and release of proinflammatory cytokines and mediators. Infected necrosis, bacteremia and organ failure are associated with intestinal barrier dysfunction in the early stages of acute pancreatitis [12]. Therefore, targeting pancreatic inflammatory responses and/or maintaining gut homeostasis would be a promising approach to treating acute pancreatitis. Although in recent years, the pharmacological tools available for pancreatitis have significantly increased in their number, this disease is an important problem worldwide [10,11]. Among the pathways involved in this pathology, oxidative stress is one of the most important [13,14]. The development of acute pancreatitis is associated with an increase in oxidative stress. Oxidative stress further increases the infiltration of inflammatory cells in the intestine, aggravates the injury of intestinal cells, destroys the barrier function of the intestines and promotes the entry of intestinal bacteria into the blood [14,15,16]. On the other hand, administration of protective factors or exposure to protective procedures inhibits the development of pancreatitis and accelerates recovery from this disease, and this effect is associated with an improvement in the redox balance [17,18,19]. Many papers have demonstrated that oxidants have a key role in the development of pancreatic tissue injury using experimental models of acute pancreatitis [20,21]. Moreover, clinical reports have indicated that oxidative stress is involved in the early phase of the disease [22]. Activated immune cells and acinar cells in injured pancreatic tissue produce oxygen free radicals, complemented by decreased total glutathione (GSH) and superoxide dismutase (SOD) levels and increased lipid peroxidation. Additionally, antioxidant administration has been reported to reduce the severity of pancreatic tissue injury and mitigate acinar cell necrosis in different models of acute pancreatitis [23,24,25]. Polyphenols and phenols in olive oil have shown important antioxidant activity [26,27]. Oleuropein is the major phenol in olive leaves and fruits [28]. It is a heterosidic ester comprising beta-glucosylated elenolic acid and hydroxytyrosol (HT). Oleuropein is metabolized to HT, which has free radical-scavenging and antioxidant activities [29]. Several in vivo experiments show the beneficial effects of HT treatment [30,31,32,33,34]. HT reduced kidney cell injury induced by hydrogen peroxide by interacting with phosphatidylinositol 3 (PI3) kinase and mitogen-activated protein (MAP) kinase [35]. In intestinal Caco-2 cells, it decreased lipid peroxidation by scavenging peroxyl radicals [36]. In human keratinocytes, HT induced heme oxygenase1 (HO-1) gene expression [37]. It has been proposed that HT acts as scavenger of superoxide anion radicals, peroxynitrite and other oxygen radicals [38]. Currently, a clinical trial is ongoing to explore the detoxifying potential of hydroxytyrosol and its effects on phase II enzyme expression (leading to xenobiotic detoxification in the liver). Among these mechanisms of resistance to inflammation and oxidative stress, the nuclear factor erythroid 2-related factor (Nrf2) is one of the most well studied both in vitro and in vivo [39,40]. Recently, various evidence has reported that HT acts by activating the Nrf2 antioxidant pathway [41,42]. The caerulein model of experimental pancreatitis is now widely used for the analysis of intracellular events in the early phase of pancreatitis. Caerulein is an analogue of the pancreatic secretagogue cholecystokinin and the administration of a supraphysiological dose of caerulein activates trypsinogen within acinar cells, by causing colocalization of digestive zymogens with lysosomal enzymes [4,43]. The model offers several advantages: it is noninvasive, since no surgical intervention affecting the bile duct or pancreatic duct is necessary, it allows easily controlled grades of injury, it is highly reproducible, and it is applicable in several animal species such as mice, rats, hamsters and dogs [44]. The administration of caerulein to mice leads to the development of acute pancreatitis. This work aimed to evaluate the effects of HT on pancreatitis, the related intestinal dysfunctions and the secondary inflammatory issues, clarifying its mechanism of action.

## 2. Materials and Methods

### 2.1. Animals

CD1 female mice (25–30 g, Envigo, Milan, Italy) were employed. The University of Messina Review Board for Animal Care (OPBA) approved the study (650/2017-PR). All animal experiments agree with the new Italian regulations (D.Lgs 2014/26), EU regulations (EU Directive 2010/63) and the ARRIVE guidelines.

### 2.2. Experimental Protocol

Caerulein hyperstimulation was employed to induce acute pancreatitis [1]. Briefly, mice were injected hourly intraperitoneally with caerulein (50 μg/kg of body weight) for 10 h. One hour after the last caerulein injection, mice were sacrificed by cervical dislocation under isoflurane inhalation anesthesia (5% in air Baxter, Rome, Italy) and serum, colon and pancreatic tissue samples were collected for further analyses.

### 2.3. Experimental Groups

Mice were randomly divided into the following groups (*n* = 30):

(1) Caerulein + vehicle (saline): mice were subjected to the caerulein injection described above and treated with vehicle (saline);
(2) Caerulein + HT (5 mg/kg): mice were subjected to the caerulein injection described above and treated intraperitoneally with hydroxytyrosol (5 mg/kg of body weight dissolved in saline);
(3) Sham + vehicle (saline): mice were subjected to the same injection but received saline instead of caerulein and were treated with vehicle (saline);
(4) Sham + HT (5 mg/kg): mice were subjected to the same injection but received saline instead of caerulein and were treated with hydroxytyrosol (5 mg/kg of body weight dissolved in saline).

The hydroxytyrosol or vehicle (saline) was given 30 min after the first caerulein injection and for two consecutive hours afterwards [45]. The volume of each solution administered was calculated according to the mice’s body weight. The tested dose was chosen based on previous studies [46].

### 2.4. Measurement of Serum Lipase, Amylase and Diamine Oxidase Activity

Fresh blood was collected and allowed to clot by leaving it undisturbed at room temperature for 20 min. Then, the clot was removed by centrifuging at 1000–2000× *g* for 10 min in a refrigerated centrifuge and the supernatant was stored at −80 °C until analysis [1]. Serum diamine oxidase (DAO), amylase and lipase activity were determined using a commercial kit (Cusabio, Houston, Texas). The DAO, amylase and lipase detection assay employed the quantitative sandwich enzyme immunoassay technique. Antibody specific for DAO or amylase or lipase was pre-coated onto a microplate. Standards and samples were pipetted into the wells and any DAO or amylase or lipase present was bound by the immobilized antibody. After removing any unbound substances, a biotin-conjugated antibody specific for DAO or amylase or lipase was added to the wells. After washing, avidin conjugated horseradish peroxidase (HRP) was added to the wells. Following a wash to remove any unbound avidin-enzyme reagent, a substrate solution was added to the wells and color developed in proportion to the amount of DAO or amylase or lipase bound in the initial step. The color development was stopped and the intensity of the color was measured (DAO detection range: 15.6–1000 mU/mL, amylase detection range: 9.38–600 mU/mL, lipase detection range: 62.5–4000 mU/mL).

### 2.5. Measurement of Antioxidant Enzyme Activity

The superoxide dismutase (SOD), glutathione peroxidase (GPx), glutathione reductase (GR) and glutathione S-transferase (GST) activity was evaluated as already described previously [47,48]. SOD activity was evaluated by quantitative sandwich enzyme immunoassay technique using a commercial kit (Cusabio, Houston, Texas). The results were expressed as U/mg of protein. For GPx activity, the absorbance was monitored at 340 nm at 37 °C for 10 min, and the results were expressed as μmol of reduced glutathione (GSH)/min/mg of protein. The GR activity was evaluated by measuring the consumption of nicotinamide adenine dinucleotide phosphate (NADPH) as a cofactor in the reduction of oxidized glutathione to reduce GSH. The results were expressed as U of GR/mg of protein. One U of enzyme activity was defined as the amount of GR that oxidizes 1 μmol of NADPH per min. The GST activity was measured using 1-chloro-2,4-dinitrobenzene (CDNB) as a substrate. The results were expressed as U of GST/mg of protein. One U of enzyme activity was defined as the amount of GST that produces 1 μmol of the conjugate of GSH with CDNB per min. The total protein concentration in the homogenate was measured using the method of Bradford.

### 2.6. Measurement of Pancreatic Lipid Peroxidation

The lipid peroxidation was evaluated by reaction between malondialdehyde (MDA), thiobarbituric acid and lipid peroxides and measured spectrophometrically at 532 nm. Results were expressed as nanomol TBA (thiobarbituric acid) reactants formed/g wet tissue [47].

### 2.7. Measurement of Pancreatic Edema

Pancreatic edema was quantified by the wet weight to dry weight ratio [1].

### 2.8. Myeloperoxidase Activity

Myeloperoxidase (MPO) activity, an indicator of PMN accumulation, was determined spectrophotometrically at 650 nm [49,50]. MPO activity was expressed in U per gram weight of wet tissue and was defined as the quantity of enzyme degrading 1 µmol of peroxide min^−1^ at 37 °C.

### 2.9. Evaluation of Cytokines and Chemokines Levels

Pancreas and colon samples were collected, homogenized and centrifuged at 10,000× *g* for 15 min at 4 °C. The supernatants were employed for evaluating tissue levels of inflammatory mediators (tumor necrosis factor alpha (TNF-α), interleukin 6 (IL-6), monocyte chemotactic protein-1 (MCP1/CCL2)) using an ELISA kit (R&D system Inc, MN, USA) [1]. The serum levels of interleukin-1 beta (IL-1β), IL-6 and TNF-α were determined by enzyme-linked immunosorbent assay kits (R&D system Inc, MN, USA) [47].

### 2.10. Histological Examination

Pancreas and colon tissue were harvested and fixed in formaldehyde solution (10% in PBS) [51]; histological sections were stained with hematoxylin and eosin and evaluated using a Leica DM6 microscope (Leica Microsystems SpA, Milan, Italy) associated with Leica LAS X Navigator software (Leica Microsystems SpA, Milan, Italy). The morphological criteria were considered as already described [52,53]. In particular, for the pancreas histology, we evaluated edema (0–4 points), acinar necrosis (0–4 points), hemorrhage and fat necrosis (0–4 points), inflammation and perivascular infiltrate (0–4 points). For the colon analysis, mucosal damage was graded from 0 to 5 according to the following criteria: grade 0, normal mucosal villi; grade 1, development of subepithelial Gruenhagen’s space at the apex of the villus, often with capillary congestion; grade 2, extension of the subepithelial space with moderate lifting of the epithelial layer from the lamina propria; grade 3, massive epithelial lifting down the sides of villi, possibly with a few denuded tips; grade 4, denuded villi with the lamina propria and dilated capillaries exposed, possibly with increased cellularity of the lamina propria; grade 5, digestion and disintegration of the lamina propria, hemorrhage and ulceration [53].

### 2.11. Immunohistochemical Analysis

Immunohistochemical analysis was performed as already described [54]. Sections were probed overnight with anti-zonula occludens (ZO) antibody (1:100; Millipore, Abingdon, UK) or anti-occludin antibody (1:100; Santa Cruz Biotechnology) or anti-intracellular adhesion molecule-1 (ICAM-1) (1:100; Santa Cruz Biotechnology) or anti-P-selectin (1:100; Santa Cruz Biotechnology). Sections were washed with phosphate-buffer saline (PBS) and incubated with peroxidase-conjugated bovine anti-mouse IgG, secondary antibody (1:2000 Jackson Immuno Research, WestGrove, Pennsylvania, USA). Specific labeling was provided with a biotin-conjugated goat anti-mouse IgG and avidin-biotin peroxidase complex (Vector Laboratories, Burlingame, CA, USA). Images were collected using a Leica DM6 (Milan, Italy) microscope. The histogram profile reports the positive pixel intensity value taken from a computer program.

### 2.12. Western Blot Analysis

Western blots were performed as described in our previous studies [55]. Pancreas and colon tissue from each mouse were suspended in an extraction’s buffer containing 0.15 µM pepstatin A, 0.2 mM phenylmethylsulfonyl fluoride (PMSF), 1 mM sodium orthovanadate and 20 µM leupeptin, homogenized at the highest setting for 2 min and centrifuged at 1000× *g* for 10 min at 4 °C. Supernatants contained the cytosolic fractions, while the pellets represented the nuclear ones. Pellets were resuspended in a second buffer containing 150 mM sodium chloride (NaCl), 1% Triton X-100, 1 mM ethylene glycol tetraacetic acid (EGTA), 10 mM tris–chloridric acid (HCl) pH 7.4, 0.2 mM PMSF, 1 mM Ethylenediaminetetraacetic acid (EDTA), 0.2 mM sodium orthovanadate and 20 µm leupeptin. After centrifugation at 4 °C for 30 min, the nuclear proteins containing the supernatants were stored at −80 °C for further analysis. Specific primary antibody anti-Nrf2 (1:1000, Santa Cruz Biotechnology) or anti-HO-1 (1:1000; Santa Cruz Biotechnology) was mixed in 1× PBS, 5% *w/v* nonfat dried milk and 0.1% Tween-20 and incubated at 4 °C overnight. After, blots were incubated with peroxidase-conjugated bovine anti-mouse IgG secondary antibody or peroxidase-conjugated goat anti-rabbit IgG (1:2000, Jackson Immuno Research) for 1 h at room temperature. To verify the equal amounts of protein, membranes were also incubated with the antibody against laminin (1:1000; Santa Cruz Biotechnology) and β-actin (1: 1000; Santa Cruz Biotechnology). Signals were identified with enhanced chemiluminescence (ECL) detection system reagent and the relative expression of the protein bands was measured by densitometry with BIORAD ChemiDocTM XRS+software (Bio-rad, Milan, Italy). A representation of blot signals was imported to analysis software (Image Quant TL, v2003).

### 2.13. Intrapancreatic Trypsin Activity

Trypsin activity was measured fluorometrically using the Rhodamine110 coupled bis-(CBZ-l-isoleucyl-l-prolyl-l-arginine amide) dihydrogen chloride, and catalytic activity in units was quantified using a microplate reader (Fluostar OPTIMA, BMG Labtech, Ortenberg, Germany). The activity was calculated and represented as U/mg of protein [56].

### 2.14. Materials

Hydroxytyrosol was purchased from Merck (Milan, Italy). All compounds used in this study, except where otherwise specified, were purchased from Sigma-Aldrich Company Ltd. (Milan, Italy).

### 2.15. Statistical Evaluation

All values in the figures and text are expressed as mean ± standard error of the mean (SEM) of N number of animals. Results were analyzed by one-way ANOVA followed by a Bonferroni post-hoc test for multiple comparisons. A *P*-value < 0.05 was considered significant. * *p* < 0.05 vs. sham + vehicle, # *p* < 0.05 vs. caerulein + vehicle, ** *p* < 0.01 vs. sham + vehicle, ## *p* < 0.01 vs. caerulein + vehicle, *** *p* < 0.001 vs. sham + vehicle, ### *p* < 0.001 vs. caerulein + vehicle.

## 3. Results

### 3.1. Hydroxytyrosol Reduces Serum Enzymes and Cytokine Alterations Induced by Acute Pancreatitis

**Figure 1:**
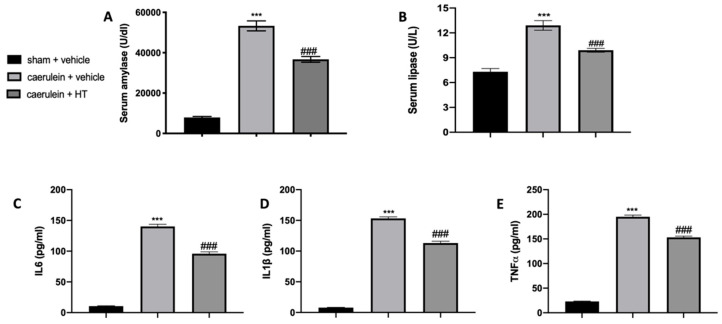
Hydroxytyrosol (HT) effect on serum parameter changes induced by caerulein administration: amylase (**A**), lipase (**B**), interleukin 6 (IL-6) (**C**), interleukin 1beta (IL-1β) (**D**) and tumor necrosis factor alpha (TNF-α) (**E**). For each analysis, *n* = 5 animals from each group were employed. A *p*-value < 0.05 was considered significant. *** *p* < 0.001 vs. sham + vehicle, ### *p* < 0.001 vs. caerulein + vehicle.

In our study, we show that HT alleviated caerulein induced pancreatitis. As first factors, we evaluated serum enzymes and inflammatory cytokine expression. Serum parameters of animals treated with vehicle showed that amylase and lipase levels were remarkably higher as compared to the sham group, while HT administration (5 mg/kg) reduced its levels (Figure 1A,B). Moreover, serum evaluation of proinflammatory cytokines showed increased levels of IL-6 (Figure 1C), IL-1β (Figure 1D) and TNF-α (Figure 1E) in vehicle-treated mice while HT treatment (5 mg/kg) reduced all these parameters. Therefore, HT reduced serum enzymes and cytokine modification induced by caerulein injection.

### 3.2. Hydroxytyrosol Restored Antioxidant Enzyme Expression and Reduced Lipid Peroxidation Induced by Acute Pancreatitis

**Figure 2:**
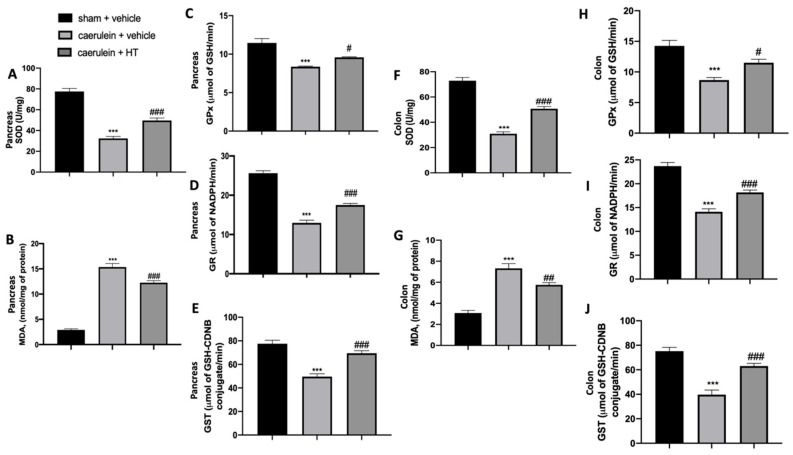
Hydroxytyrosol (HT) effect on oxidative stress induced by caerulein administration: pancreas tissue: superoxide dismutase (SOD) (**A**), malondialdehyde (MDA) (**B**), glutathione peroxidase (GPx) (**C**), glutathione reductase (GR) (**D**), glutathione S-transferase (GST) (**E**), colon tissue: SOD (**F**), MDA (**G**), GPx (**H**), GR (**I**), GST (**J**). For each analysis, *n* = 5 animals from each group were employed. A *p*-value < 0.05 was considered significant. # *p* < 0.05 vs. caerulein + vehicle, *** *p* < 0.001 vs. sham + vehicle, ### *p* < 0.001 vs. caerulein + vehicle.

In the inflammatory response to caerulein injection, oxidative stress is strictly involved. We measured SOD activity and MDA levels to assess pancreas and colon injuries. We showed that HT treatment (5 mg/kg) significantly increased SOD activity (Figure 2A,F) and reduced MDA levels (Figure 2B,G) both in pancreas and colon tissue as compared to vehicle-treated mice. Caerulein injection reduced GPx (Figure 2C,H), GR (Figure 2D,I) and GST (Figure 2E,J) compared to the sham animals. HT treatment (5 mg/kg) upregulated all these parameters. In conclusion, HT increased antioxidant enzyme expression and reduced lipid peroxidation induced by caerulein injection.

### 3.3. Hydroxytyrosol Enhanced Nrf2/HO-1 Expression in Pancreatic and Colonic Tissue

**Figure 3:**
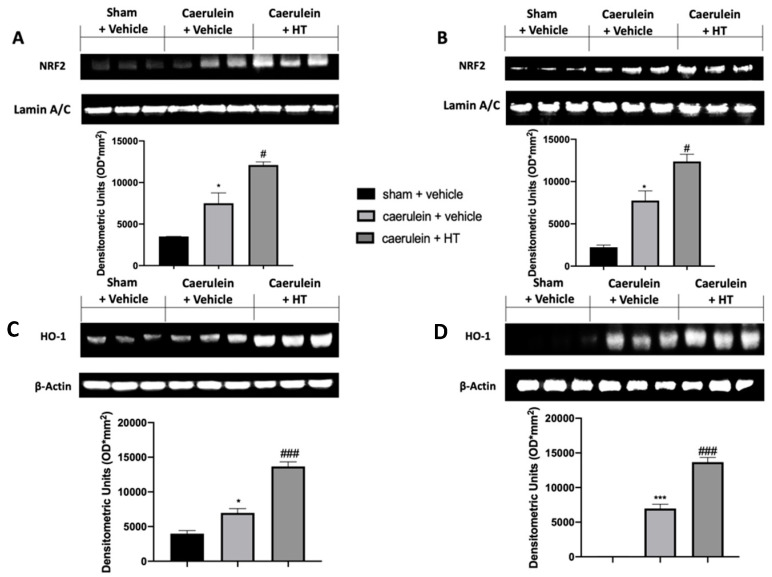
Hydroxytyrosol (HT) effect on nuclear factor erythroid 2-related factor 2 (Nrf2)/ heme oxygenase 1 (HO-1) expression: Western blot analysis of: Nrf2 from pancreatic tissue (**A**), Nrf2 from colonic tissue (**B**), HO-1 from pancreatic tissue (**C**), HO-1 from colonic tissue (**D**). For each analysis, *n* = 5 animals from each group were employed. A *p*-value < 0.05 was considered significant. * *p* < 0.05 vs. sham + vehicle, # *p* < 0.05 vs. caerulein + vehicle, *** *p* < 0.001 vs. sham + vehicle, ### *p* < 0.001 vs. caerulein + vehicle.

The Nrf2/HO-1 system is one of the crucial antioxidant pathways induced by pancreatitis. We evaluated the expression levels of these proteins in the injured tissues. Nrf2 expression levels, monitored by Western blotting in both pancreas (Figure 3A) and colon (Figure 3B) tissue, were increased in both tissues harvested from vehicle-treated mice, as compared to the sham basal levels. HT administration (5 mg/kg) increased Nrf2 levels in both tissues compared to the vehicle group. Western blot analysis also showed basal expression of HO-1 in both pancreas (Figure 3C) and colon (Figure 3D) tissue of sham animals, while vehicle-treated mice displayed increased levels. HO-1 expression was upregulated in both pancreas and colon tissues of HT (5 mg/kg) administered animals. Thus, our results showed that HT could manage the Nrf2/HO-1 pathway.

### 3.4. Hydroxytyrosol Ameliorated Pancreas Histological Injury and Cytokine Expression Changes Induced by Pancreatitis

**Figure 4:**
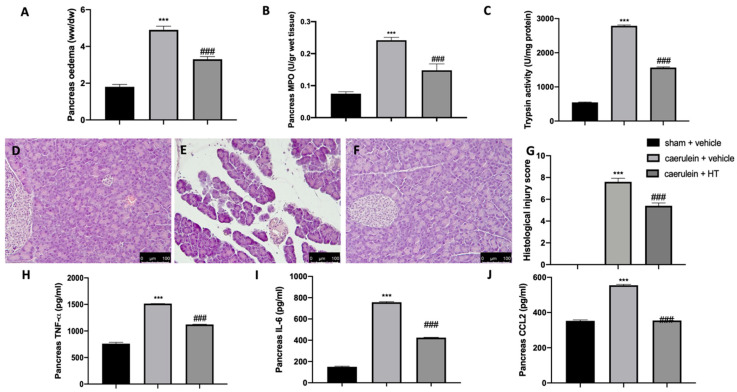
Hydroxytyrosol (HT) effect on pancreas injury induced by caerulein administration: pancreaetic edema (**A**), myeloperoxidase (MPO) activity (**B**), trypsin activity (**C**), pancreas histological analysis: sham + vehicle (**D**), caerulein + vehicle (**E**), caerulein + HT (**F**), histological injury score (**G**), pancreas cytokine expression: tumor necrosis factor alpha (TNF-α) (**H**), interleukin 6 (IL-6) (**I**), monocyte chemotactic protein-1 (MCP1/CCL2) (**J**). For each analysis, *n* = 5 animals from each group were employed. A *p*-value < 0.05 was considered significant. *** *p* < 0.001 vs. sham + vehicle, ### *p* < 0.001 vs. caerulein + vehicle. Scale bar 100 μm.

It is well known that the administration of caerulein to mice leads to the development of severe acute necrotizing pancreatitis, in contrast to rats, where caerulein leads to the development of mild edematous acute pancreatitis. Our data showed that caerulein administration increased pancreatic edema (Figure 4A) and pancreatic MPO (Figure 4B) and trypsin (Figure 4C) activity. HT administration (5 mg/kg) showed reduced edema, neutrophils and trypsin activity. Histological analysis of pancreatic tissue further confirmed the beneficial effect of HT (5 mg/kg), shown by decreased inflammatory cell accumulation and ameliorated cellular morphology acinar necrosis (Figure 4F,G) induced by caerulein injection (Figure 4E,G). No histological changes were detected in sham animals (Figure 4D,G and Figure S1). Additionally, HT (5 mg/kg) decreased pancreatic TNF-α (Figure 4H), IL-6 (Figure 4I) and MCP1/CCL2 levels (Figure 4J). These data showed that HT improved pancreas histological injury and cytokine release induced by caerulein injection.

### 3.5. Hydroxytyrosol Attenuates Intestinal Injury Associated with Acute Pancreatitis

**Figure 5:**
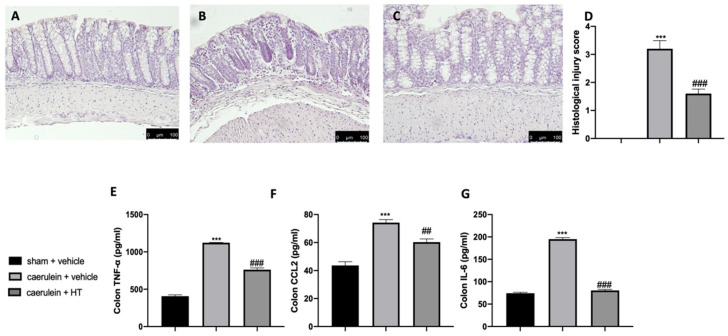
Hydroxytyrosol (HT) effect on colon inflammation: colon histological analysis: sham + vehicle (**A**), caerulein + vehicle (**B**), caerulein + HT(**C**), histological injury score (**D**), colon cytokine expression: tumor necrosis factor alpha (TNF-α) (**E**), monocyte chemotactic protein-1 (MCP1/CCL2) (**F**), interleukin 6 (IL-6) (**G**). For the analyses, *n* = 5 animals from each group were employed. A *p*-value < 0.05 was considered significant. ## *p* < 0.01 vs. caerulein + vehicle, *** *p* < 0.001 vs. sham + vehicle, ### *p* < 0.001 vs. caerulein + vehicle.

Acute pancreatitis is further characterized by dysregulated gut homeostasis. In particular, histological analysis of colon tissue showed that vehicle-treated mice displayed tissue inflammation and cellular recruitment (Figure 5B,D) as compared to sham animals (Figure 5A,D and Figure S1). HT administration (5 mg/kg) reduced this inflammation (Figure 5C,D). Moreover, HT (5 mg/kg) reduced intestinal proinflammatory cytokine expression, including TNF-α (Figure 5E), MCP1/CCL2 (Figure 5F), and IL-6 (Figure 5G). These data showed that HT improved intestinal histological injury and cytokine release induced by caerulein injections.

### 3.6. Hydroxytyrosol Reduces Mast Cell Recruitment in Pancreas and Colon Tissue Associated with Acute Pancreatitis

**Figure 6:**
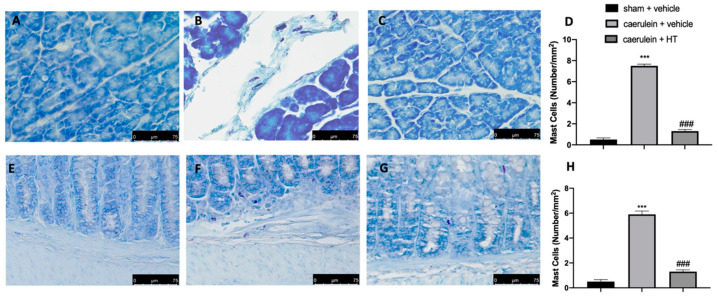
Hydroxytyrosol (HT) effect on mast cell infiltration: toluidine blue staining of pancreas tissue: sham + vehicle (**A**), caerulein + vehicle (**B**), caerulein + HT (5 mg/kg) (**C**), mast cell count (**D**), toluidine blue staining of colon tissue: sham + vehicle (**E**), caerulein + vehicle (**F**), caerulein + HT (5 mg/kg) (**G**), mast cell count (**H**). For the analyses, *n* = 5 animals from each group were employed. A *p*-value < 0.05 was considered significant. *** *p* < 0.001 vs. sham + vehicle, ### *p* < 0.001 vs. caerulein + vehicle.

Thereafter, we evaluated inflammatory cell recruitment to the lesion sites. Toluidine blue staining showed increased mast cell recruitment both in pancreas and colon tissue collected from vehicle-treated mice (Figure 6B,D,F,H, respectively), as compared to the tissue from the sham group (Figure 6A,D,E,H, respectively). HT administration (5 mg/kg) reduced mast cell infiltration in both inflamed tissues (Figure 6C,D,G,H, respectively). Thus, HT reduced mast cell recruitment in both pancreas and colon tissue induced by caerulein injection.

### 3.7. Hydroxytyrosol Decreases Adhesion Molecule Expression Associated with Acute Pancreatitis

**Figure 7:**
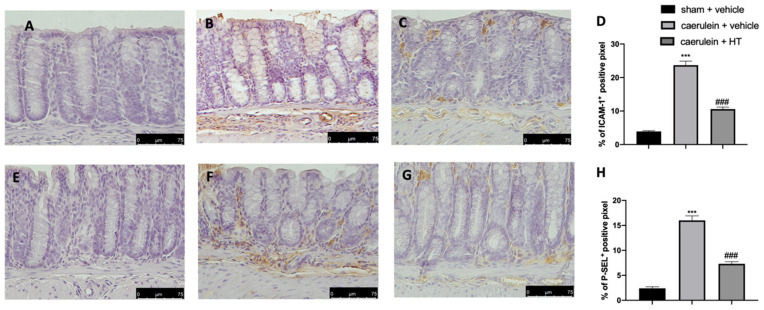
Hydroxytyrosol (HT) effect on intercellular adhesion molecule-1 (ICAM-1) and P-selectin expression: immunohistochemical analysis of ICAM-1 of sham + vehicle (**A**), caerulein + vehicle (**B**) and caerulein + HT (5 mg/kg) (**C**) administration. Graphical quantification of ICAM-1 expression (**D**). Immunohistochemical analysis of P-selectin of sham + vehicle (**E**), caerulein + vehicle (**F**) and caerulein + HT (5 mg/kg) (**G**) administration. Graphical quantification of P-selectin expression (**H**). For the analyses, *n* = 5 animals from each group were employed. A *p*-value < 0.05 was considered significant. *** *p* < 0.001 vs. sham + vehicle, ### *p* < 0.001 vs. caerulein + vehicle.

The selectin family and ICAMs are expressed on the membranes of leukocytes and endothelial cells and play roles in leukocyte adhesion and rolling in blood vessels. They are involved in the pathogenesis of systemic inflammation and multiple organ failure in acute pancreatitis [57]; therefore, immunohistochemical analysis to evaluate their expression was performed. Increased adhesion molecule expression (ICAM-1 and P-selectin) was detected in samples collected from vehicle-treated mice (Figure 7B,D,F,H, respectively), as compared to sham samples (Figure 7A,D,E,H, respectively). HT administration (5 mg/kg) reduced both ICAM-1 (Figure 7C,D) and P-selectin (Figure 7G,H) expression in colon tissue.

### 3.8. Hydroxytyrosol Preserves the Integrity of the Gut Barrier

**Figure 8:**
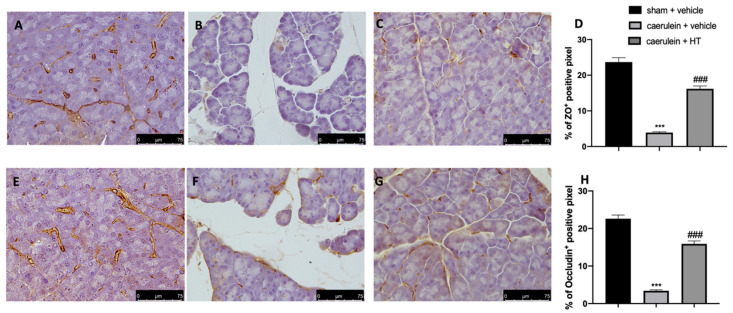
Hydroxytyrosol (HT) effect on zonula occludens (ZO) and occludin expression in pancreatic tissue: immunohistochemical analysis of ZO of sham + vehicle (**A**), caerulein + vehicle (**B**) and caerulein + HT (5 mg/kg) (**C**) administration. Graphical quantification of ZO expression (D). Immunohistochemical analysis of occludin of sham + vehicle (**E**), caerulein + vehicle (**F**) and caerulein + HT (5 mg/kg) (**G**) administration. Graphical quantification occludin expression (H). For the analyses, *n* = 5 animals from each group were employed. A *p*-value < 0.05 was considered significant. *** *p* < 0.001 vs. sham + vehicle, ### *p* < 0.001 vs. caerulein + vehicle.

**Figure 9:**
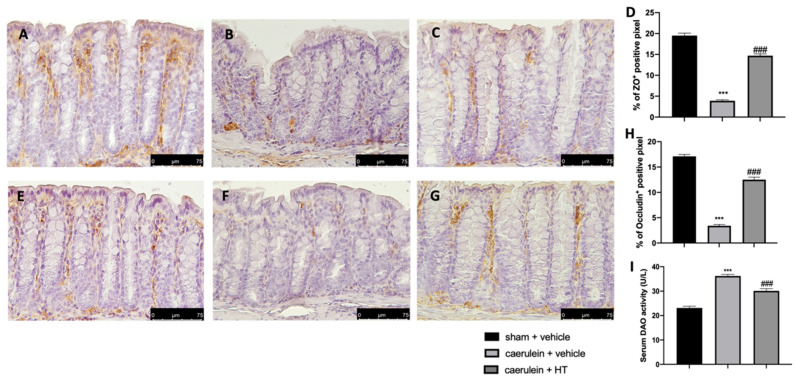
Hydroxytyrosol (HT) effect on zonula occludens (ZO) and occludin expression in colonic tissue and DAO activity: immunohistochemical analysis of ZO of sham + vehicle (**A**), caerulein + vehicle (**B**) and caerulein + HT (5 mg/kg) (**C**) administration. Graphical quantification of ZO expression (**D**). Immunohistochemical analysis of occludin of sham + vehicle (**E**), caerulein + vehicle (**F**) and caerulein + HT (5 mg/kg) (**G**) administration. Graphical quantification occludin expression (**H**), serum diamine oxidase (DAO) activity (**I**). For the analyses, *n* = 5 animals from each group were employed. A *p*-value <0.05 was considered significant. *** *p* < 0.001 vs. sham + vehicle, ### *p* < 0.001 vs. caerulein + vehicle.

Loosening of cell–cell adhesion between pancreatic acinar cells and/or endothelial cells increases solute permeability, resulting in interstitial edema, which promotes inflammatory cell migration and disrupts tissue structure [57]. Immunohistochemical analysis of tight junction expression was performed in both pancreas and colon tissue. Tissue collected from vehicle-treated mice showed reduced ZO (Figure 8B,D and Figure 9B,D, respectively) and occludin (Figure 8F,H and Figure 9F,H) expression as compared to sham tissues (Figure 8A,D; Figure 9A,D; Figure 8E,H; Figure 9E,H). HT administration (5 mg/kg) was able to restore ZO and occludin expression in both pancreas (Figure 8C,D,G,H) and colon (Figure 9C,D,G,H) tissue. Additionally, HT (5 mg/kg) improved intestinal barrier function, as displayed by serum DAO activity (Figure 9I).

## 4. Discussion

Pancreatitis is a severe disease with multifaceted underlying mechanisms which are still poorly understood. This disease is associated with comorbidities and mortality [58,59]. From a molecular point of view, pancreatitis is characterized by inflammatory processes [60] and oxidative stress increase [48], while one of the most frequent extrapancreatic complications is gut dysfunction [61]. The lack of effective therapeutic choices necessitates increasing research for effective therapy for the management of pancreatitis. HT showed evident anti-inflammatory, antithrombotic and antiatherogenic properties [62,63,64]. It is also able to contrast hyperglycemia, insulin resistance and metabolic syndrome, improving endothelial function and hemostatic and lipid profiles [65] and decreasing oxidative stress [66,67]. In addition, HT showed further positive health effects such as antisteatotic and pro-autophagic properties and improved mitochondrial function [68]. Our findings show that HT can protect from acute pancreatitis and gut comorbidity by the following results: HT reduced serum and cytokine activation, lipid peroxidation and oxidative stress, pancreas and colon histological changes, tight junction disruption and inflammatory cell recruitment in the inflamed tissues. Crucially, these effects were mediated by the NRF2 pathway. During pancreatitis, serum amylase, lipase and cytokine, such as IL6, IL-1β and TNF-α, levels are increased. Additionally, there is early intra-acinar cell activation of inactive zymogens into their active forms. Following this early activation, a trypsin cascade occurs in the gland, which leads to the auto-digestion of acinar cells [4]. HT administration reduced serum enzyme overexpression and cytokines as well. Moreover, HT treatment reduced trypsin activity, showing the ability to manage the intrapancreatic protease activation induced by acute pancreatitis. Under homeostatic conditions, tissues have several endogenous antioxidant enzymes, including SOD and glutathione, which act as ROS scavengers, preventing lipid peroxidation [69]. In this pathology, free oxygen radicals are overproduced, shifting the endogenous oxidant/antioxidant balance. HT administration was able to restore this equilibrium to the homeostatic condition in both pancreas and colon tissue, as shown by the increased SOD, GPx, GR and GST activity, while MDA levels were decreased. HT was able to ameliorate pancreas and intestinal oxidative damage. The Nrf2 and NF-kB pathways are closely related in the oxidative stress [70] and inflammatory [40] responses, respectively. Nrf2 is a transcription factor usually localized in the cytosol and regulated by Kelch-like ECH-associated protein 1 (Keap1). ROS generation induces Nrf2 translocation into the nucleus, where it modulates the transcription of multiple target genes, including phase II detoxification enzymes such as heme oxygenase 1 (HO-1) [71]. Recent findings showed that mice lacking NFR2 displayed more severe pathological changes in both pancreatic and gut tissue [72]. Here, we showed that HT was able to fight the oxidative stress induced by caerulein administration by activating the NRF2 pathway and enhancing HO-1 expression. These results are well in line with the pancreas and intestinal morphological changes detected. HT attenuated acinar necrosis and pancreatic edema and MPO activity, an activated neutrophils biomarker. From a histological point of view, HT protected pancreas tissue from the pathological changes induced by caerulein. Additionally, injured acinar cells produce cytokines and chemokines to initiate inflammatory responses [73]. HT reduced TNF-α, IL6 and MCP1/CCL2 overexpression in the pancreas. Several studies suggest that inflammatory cell and cytokine infiltration are two critical events in pancreatitis induced distant organ injuries, leading to systemic and local complications [74]. Our results showed that HT administration was able to reduce colon inflammation, swelling, inflammatory cell infiltration and cytokine and chemokine overexpression in this tissue. Recruitment of inflammatory cells has a key role in the disease [75]. Our data suggested that HT limited the recruitment of mast cells in the pancreas and colon during pancreatitis. The inflammatory cells’ infiltration into the inflamed tissues is mediated by the overexpression of the adhesion molecules [50,76]. They play important roles in cell migration, proliferation and signal transduction, as well as in development and tissue repair. HT administration was able to reduce ICAM-1 and P-selectin overexpression in colon tissue. Additionally, the gut barrier dysfunction associated with pancreatitis is characterized by downregulation of the expression of tight junction proteins, impaired intestinal motility, bacterial translocation and increased intestinal permeability [77]. The damaged intestinal barrier allowed organisms to move through the bloodstream, producing multiple organ failure [78,79]. Consequently, maintaining intestinal integrity decreases morbidity associated with pancreatitis. DAO, synthetized by the epithelial cells of the intestinal villi, is a biomarker of the integrity of the gut barrier. The increased serum levels of DAO enzymes and the damaged tight junctions indicated this tissue injury and intestinal barrier dysfunction [61]. These parameters were considerably reduced in animals administered HT treatment.

## 5. Conclusions

Collectively, our data demonstrate that HT, by reducing inflammatory cell infiltration, cytokine overexpression, oxidative stress and the NRF2 pathway, counteracted pancreatic damage and associated intestinal injury during pancreatitis. Conclusively, we propose HT as potential strategy for pancreatitis and intestinal comorbidity treatment.

## Supplementary Materials

The following are available online at https://www.mdpi.com/2076-3921/9/9/781/s1, Figure S1: Hematoxylin and eosin staining of pancreas and colon tissue.

## References

[1] Pan X., Fang X., Wang F., Li H., Niu W., Liang W., Wu C., Li J., Tu X., Pan L.L. (2019). Butyrate ameliorates caerulein-induced acute pancreatitis and associated intestinal injury by tissue-specific mechanisms. Br. J. Pharmacol..

[2] Steer M.L., Meldolesi J., Figarella C. (1984). Pancreatitis. The role of lysosomes. Dig. Dis. Sci..

[3] Saito I., Hashimoto S., Saluja A., Steer M.L., Meldolesi J. (1987). Intracellular transport of pancreatic zymogens during caerulein supramaximal stimulation. Am. J. Physiol..

[4] Frossard J.L., Pastor C.M. (2002). Experimental acute pancreatitis: New insights into the pathophysiology. Front. Biosci..

[5] Xiao A.Y., Tan M.L., Wu L.M., Asrani V.M., Windsor J.A., Yadav D., Petrov M.S. (2016). Global incidence and mortality of pancreatic diseases: A systematic review, meta-analysis, and meta-regression of population-based cohort studies. Lancet Gastroenterol. Hepatol..

[6] Uhl W., Warshaw A., Imrie C., Bassi C., McKay C.J., Lankisch P.G., Carter R., Di Magno E., Banks P.A., Whitcomb D.C. (2002). International Association of, P., IAP Guidelines for the Surgical Management of Acute Pancreatitis. Pancreatology.

[7] Watanabe T., Kudo M., Strober W. (2017). Immunopathogenesis of pancreatitis. Mucosal Immunol..

[8] Sendler M., Dummer A., Weiss F.U., Kruger B., Wartmann T., Scharffetter-Kochanek K., van Rooijen N., Malla S.R., Aghdassi A., Halangk W. (2013). Tumour necrosis factor alpha secretion induces protease activation and acinar cell necrosis in acute experimental pancreatitis in mice. Gut.

[9] He Y., Wu C., Li J., Li H., Sun Z., Zhang H., de Vos P., Pan L.L., Sun J. (2017). Inulin-Type Fructans Modulates Pancreatic-Gut Innate Immune Responses and Gut Barrier Integrity during Experimental Acute Pancreatitis in a Chain Length-Dependent Manner. Front. Immunol..

[10] Lu G., Tong Z., Ding Y., Liu J., Pan Y., Gao L., Tu J., Wang Y., Liu G., Li W. (2016). Aspirin Protects against Acinar Cells Necrosis in Severe Acute Pancreatitis in Mice. Biomed. Res. Int..

[11] Lankisch P.G., Apte M., Banks P.A. (2015). Acute pancreatitis. Lancet.

[12] Ammori B.J. (2003). Role of the gut in the course of severe acute pancreatitis. Pancreas.

[13] Finkel T., Holbrook N.J. (2000). Oxidants, oxidative stress and the biology of ageing. Nature.

[14] Tsai K., Wang S.S., Chen T.S., Kong C.W., Chang F.Y., Lee S.D., Lu F.J. (1998). Oxidative stress: An important phenomenon with pathogenetic significance in the progression of acute pancreatitis. Gut.

[15] Tian R., Tan J.T., Wang R.L., Xie H., Qian Y.B., Yu K.L. (2013). The role of intestinal mucosa oxidative stress in gut barrier dysfunction of severe acute pancreatitis. Eur. Rev. Med. Pharmacol. Sci..

[16] Siriwardena A.K., Mason J.M., Balachandra S., Bagul A., Galloway S., Formela L., Hardman J.G., Jamdar S. (2007). Randomised, double blind, placebo controlled trial of intravenous antioxidant (n-acetylcysteine, selenium, vitamin C) therapy in severe acute pancreatitis. Gut.

[17] Robles L., Vaziri N.D., Ichii H. (2013). Role of Oxidative Stress in the Pathogenesis of Pancreatitis: Effect of Antioxidant Therapy. Pancreat. Disord. Ther..

[18] Schulz H.U., Niederau C., Klonowski-Stumpe H., Halangk W., Luthen R., Lippert H. (1999). Oxidative stress in acute pancreatitis. Hepatogastroenterology.

[19] Perez S., Pereda J., Sabater L., Sastre J. (2015). Redox signaling in acute pancreatitis. Redox Biol..

[20] Xie Q., Fei M., Fa Z., Wang L., Wang J., Zhang Y., Wang J., Deng X. (2017). Methane-rich saline alleviates cerulein-induced acute pancreatitis by inhibiting inflammatory response, oxidative stress and pancreatic apoptosis in mice. Int. Immunopharmacol..

[21] Nuhn P., Mitkus T., Ceyhan G.O., Kunzli B.M., Bergmann F., Fischer L., Giese N., Friess H., Berberat P.O. (2013). Heme oxygenase 1-generated carbon monoxide and biliverdin attenuate the course of experimental necrotizing pancreatitis. Pancreas.

[22] Esrefoglu M. (2012). Experimental and clinical evidence of antioxidant therapy in acute pancreatitis. World J. Gastroenterol..

[23] Yang F., Xie J., Wang W., Xie Y., Sun H., Jin Y., Xu D., Chen B., Andersson R., Zhou M. (2014). Regional arterial infusion with lipoxin A4 attenuates experimental severe acute pancreatitis. PLoS ONE.

[24] Dong Z., Shang H., Chen Y.Q., Pan L.L., Bhatia M., Sun J. (2016). Sulforaphane Protects Pancreatic Acinar Cell Injury by Modulating Nrf2-Mediated Oxidative Stress and NLRP3 Inflammatory Pathway. Oxid. Med. Cell. Longev..

[25] Yu Q.H., Zhang P.X., Liu Y., Liu W., Yin N. (2016). Hyperbaric oxygen preconditioning protects the lung against acute pancreatitis induced injury via attenuating inflammation and oxidative stress in a nitric oxide dependent manner. Biochem. Biophys. Res. Commun..

[26] Medina I., Satue-Gracia M.T., German J.B., Frankel E.N. (1999). Comparison of natural polyphenol antioxidants from extra virgin olive oil with synthetic antioxidants in tuna lipids during thermal oxidation. J. Agric. Food Chem..

[27] Pellegrini N., Visioli F., Buratti S., Brighenti F. (2001). Direct analysis of total antioxidant activity of olive oil and studies on the influence of heating. J. Agric. Food Chem..

[28] Zoidou E., Melliou E., Gikas E., Tsarbopoulos A., Magiatis P., Skaltsounis A.L. (2010). Identification of Throuba Thassos, a traditional Greek table olive variety, as a nutritional rich source of oleuropein. J. Agric. Food Chem..

[29] Ortega-Garcia F., Blanco S., Peinado M.A., Peragon J. (2008). Polyphenol oxidase and its relationship with oleuropein concentration in fruits and leaves of olive (Olea europaea) cv. ‘Picual’ trees during fruit ripening. Tree Physiol..

[30] Granados-Principal S., Quiles J.L., Ramirez-Tortosa C., Camacho-Corencia P., Sanchez-Rovira P., Vera-Ramirez L., Ramirez-Tortosa M.C. (2011). Hydroxytyrosol inhibits growth and cell proliferation and promotes high expression of sfrp4 in rat mammary tumours. Mol. Nutr. Food Res..

[31] Mateos R., Martinez-Lopez S., Arevalo G.B., Amigo-Benavent M., Sarria B., Bravo-Clemente L. (2016). Hydroxytyrosol in functional hydroxytyrosol-enriched biscuits is highly bioavailable and decreases oxidised low density lipoprotein levels in humans. Food Chem..

[32] Granados-Principal S., El-Azem N., Pamplona R., Ramirez-Tortosa C., Pulido-Moran M., Vera-Ramirez L., Quiles J.L., Sanchez-Rovira P., Naudi A., Portero-Otin M. (2014). Hydroxytyrosol ameliorates oxidative stress and mitochondrial dysfunction in doxorubicin-induced cardiotoxicity in rats with breast cancer. Biochem. Pharmacol..

[33] Zheng A., Li H., Cao K., Xu J., Zou X., Li Y., Chen C., Liu J., Feng Z. (2015). Maternal hydroxytyrosol administration improves neurogenesis and cognitive function in prenatally stressed offspring. J. Nutr. Biochem..

[34] Bullon P., Quiles J.L., Morillo J.M., Rubini C., Goteri G., Granados-Principal S., Battino M., Ramirez-Tortosa M. (2009). Gingival vascular damage in atherosclerotic rabbits: Hydroxytyrosol and squalene benefits. Food Chem. Toxicol..

[35] Incani A., Deiana M., Corona G., Vafeiadou K., Vauzour D., Dessi M.A., Spencer J.P. (2010). Involvement of ERK, Akt and JNK signalling in H2O2-induced cell injury and protection by hydroxytyrosol and its metabolite homovanillic alcohol. Mol. Nutr Food Res..

[36] Deiana M., Corona G., Incani A., Loru D., Rosa A., Atzeri A., Paola Melis M., Assunta Dessi M. (2010). Protective effect of simple phenols from extravirgin olive oil against lipid peroxidation in intestinal Caco-2 cells. Food Chem. Toxicol..

[37] Sgarbossa A., Dal Bosco M., Pressi G., Cuzzocrea S., Dal Toso R., Menegazzi M. (2012). Phenylpropanoid glycosides from plant cell cultures induce heme oxygenase 1 gene expression in a human keratinocyte cell line by affecting the balance of NRF2 and BACH1 transcription factors. Chem. Biol. Interact..

[38] Umeno A., Horie M., Murotomi K., Nakajima Y., Yoshida Y. (2016). Antioxidative and Antidiabetic Effects of Natural Polyphenols and Isoflavones. Molecules.

[39] Peng S., Zhang B., Yao J., Duan D., Fang J. (2015). Dual protection of hydroxytyrosol, an olive oil polyphenol, against oxidative damage in PC12 cells. Food Funct..

[40] Kim S., Indu Viswanath A.N., Park J.H., Lee H.E., Park A.Y., Choi J.W., Kim H.J., Londhe A.M., Jang B.K., Lee J. (2020). Nrf2 activator via interference of Nrf2-Keap1 interaction has antioxidant and anti-inflammatory properties in Parkinson’s disease animal model. Neuropharmacology.

[41] Leri M., Scuto M., Ontario M.L., Calabrese V., Calabrese E.J., Bucciantini M., Stefani M. (2020). Healthy Effects of Plant Polyphenols: Molecular Mechanisms. Int. J. Mol. Sci..

[42] Montoya T., Aparicio-Soto M., Castejon M.L., Rosillo M.A., Sanchez-Hidalgo M., Begines P., Fernandez-Bolanos J.G., Alarcon-de-la-Lastra C. (2018). Peracetylated hydroxytyrosol, a new hydroxytyrosol derivate, attenuates LPS-induced inflammatory response in murine peritoneal macrophages via regulation of non-canonical inflammasome, Nrf2/HO1 and JAK/STAT signaling pathways. J. Nutr. Biochem..

[43] Halangk W., Lerch M.M., Brandt-Nedelev B., Roth W., Ruthenbuerger M., Reinheckel T., Domschke W., Lippert H., Peters C., Deussing J. (2000). Role of cathepsin B in intracellular trypsinogen activation and the onset of acute pancreatitis. J. Clin. Investig..

[44] Lerch M.M., Adler G. (1994). Experimental animal models of acute pancreatitis. Int. J. Pancreatol..

[45] Lin Z.S., Ku C.F., Guan Y.F., Xiao H.T., Shi X.K., Wang H.Q., Bian Z.X., Tsang S.W., Zhang H.J. (2016). Dihydro-Resveratrol Ameliorates Lung Injury in Rats with Cerulein-Induced Acute Pancreatitis. Phytother. Res..

[46] Silva S., Sepodes B., Rocha J., Direito R., Fernandes A., Brites D., Freitas M., Fernandes E., Bronze M.R., Figueira M.E. (2015). Protective effects of hydroxytyrosol-supplemented refined olive oil in animal models of acute inflammation and rheumatoid arthritis. J. Nutr. Biochem..

[47] Li Y., Pan Y., Gao L., Zhang J., Xie X., Tong Z., Li B., Li G., Lu G., Li W. (2018). Naringenin Protects against Acute Pancreatitis in Two Experimental Models in Mice by NLRP3 and Nrf2/HO-1 Pathways. Mediat. Inflamm..

[48] Deng W., Abliz A., Xu S., Sun R., Guo W., Shi Q., Yu J., Wang W. (2016). Severity of pancreatitisassociated intestinal mucosal barrier injury is reduced following treatment with the NADPH oxidase inhibitor apocynin. Mol. Med. Rep..

[49] Fusco R., D’amico R., Cordaro M., Gugliandolo E., Siracusa R., Peritore A.F., Crupi R., Impellizzeri D., Cuzzocrea S., Di Paola R. (2018). Absence of formyl peptide receptor 1 causes endometriotic lesion regression in a mouse model of surgically-induced endometriosis. Oncotarget.

[50] Siracusa R., Fusco R., Peritore A.F., Cordaro M., D’Amico R., Genovese T., Gugliandolo E., Crupi R., Smeriglio A., Mandalari G. (2020). The Antioxidant and Anti-Inflammatory Properties of Anacardium occidentale L. Cashew Nuts in a Mouse Model of Colitis. Nutrients.

[51] Cordaro M., Impellizzeri D., Siracusa R., Gugliandolo E., Fusco R., Inferrera A., Esposito E., Di Paola R., Cuzzocrea S. (2017). Effects of a co-micronized composite containing palmitoylethanolamide and polydatin in an experimental model of benign prostatic hyperplasia. Toxicol. Appl. Pharmacol..

[52] Schmidt J., Rattner D.W., Lewandrowski K., Compton C.C., Mandavilli U., Knoefel W.T., Warshaw A.L. (1992). A better model of acute pancreatitis for evaluating therapy. Ann. Surg..

[53] Deng Y.Y., Shamoon M., He Y., Bhatia M., Sun J. (2016). Cathelicidin-related antimicrobial peptide modulates the severity of acute pancreatitis in mice. Mol. Med. Rep..

[54] Gugliandolo E., Fusco R., Biundo F., D’Amico R., Benedetto F., Di Paola R., Cuzzocrea S. (2017). Palmitoylethanolamide and Polydatin combination reduces inflammation and oxidative stress in vascular injury. Pharmacol. Res..

[55] Gugliandolo E., Fusco R., D’Amico R., Militi A., Oteri G., Wallace J.L., Di Paola R., Cuzzocrea S. (2018). Anti-inflammatory effect of ATB-352, a H2S -releasing ketoprofen derivative, on lipopolysaccharide-induced periodontitis in rats. Pharmacol. Res..

[56] Malla S.R., Krueger B., Wartmann T., Sendler M., Mahajan U.M., Weiss F.U., Thiel F.G., De Boni C., Gorelick F.S., Halangk W. (2020). Early trypsin activation develops independently of autophagy in caerulein-induced pancreatitis in mice. Cell. Mol. Life Sci..

[57] Sato T., Shibata W., Maeda S. (2019). Adhesion molecules and pancreatitis. J. Gastroenterol..

[58] Zerem E. (2014). Treatment of severe acute pancreatitis and its complications. World J. Gastroenterol..

[59] Shah A.P., Mourad M.M., Bramhall S.R. (2018). Acute pancreatitis: Current perspectives on diagnosis and management. J. Inflamm. Res..

[60] Bonior J., Warzecha Z., Ceranowicz P., Gajdosz R., Pierzchalski P., Kot M., Leja-Szpak A., Nawrot-Porabka K., Link-Lenczowski P., Pedziwiatr M. (2017). Capsaicin-Sensitive Sensory Nerves Are Necessary for the Protective Effect of Ghrelin in Cerulein-Induced Acute Pancreatitis in Rats. Int. J. Mol. Sci..

[61] Zhang J.W., Zhang G.X., Chen H.L., Liu G.L., Owusu L., Wang Y.X., Wang G.Y., Xu C.M. (2015). Therapeutic effect of Qingyi decoction in severe acute pancreatitis-induced intestinal barrier injury. World J. Gastroenterol..

[62] Scoditti E., Capurso C., Capurso A., Massaro M. (2014). Vascular effects of the Mediterranean diet—Part II: Role of omega-3 fatty acids and olive oil polyphenols. Vasc. Pharmacol..

[63] Richard N., Arnold S., Hoeller U., Kilpert C., Wertz K., Schwager J. (2011). Hydroxytyrosol is the major anti-inflammatory compound in aqueous olive extracts and impairs cytokine and chemokine production in macrophages. Planta Medica.

[64] Scoditti E., Nestola A., Massaro M., Calabriso N., Storelli C., De Caterina R., Carluccio M.A. (2014). Hydroxytyrosol suppresses MMP-9 and COX-2 activity and expression in activated human monocytes via PKCα and PKCβ1 inhibition. Atherosclerosis.

[65] Fuentes F., Lopez-Miranda J., Perez-Martinez P., Jimenez Y., Marin C., Gomez P., Fernandez J., Caballero J., Delgado-Lista J., Perez-Jimenez F. (2008). Chronic effects of a high-fat diet enriched with virgin olive oil and a low-fat diet enriched with α-linolenic acid on postprandial endothelial function in healthy men. Br. J. Nutr..

[66] Bigagli E., Cinci L., Paccosi S., Parenti A., D’Ambrosio M., Luceri C. (2017). Nutritionally relevant concentrations of resveratrol and hydroxytyrosol mitigate oxidative burst of human granulocytes and monocytes and the production of pro-inflammatory mediators in LPS-stimulated RAW 264.7 macrophages. Int. Immunopharmacol..

[67] Crupi R., Palma E., Siracusa R., Fusco R., Gugliandolo E., Cordaro M., Impellizzeri D., De Caro C., Calzetta L., Cuzzocrea S. (2020). Protective Effect of Hydroxytyrosol Against Oxidative Stress Induced by the Ochratoxin in Kidney Cells: In vitro and in vivo Study. Front. Vet. Sci..

[68] Echeverría F., Ortiz M., Valenzuela R., Videla L.A. (2017). Hydroxytyrosol and cytoprotection: A projection for clinical interventions. Int. J. Mol. Sci..

[69] Ju K.D., Lim J.W., Kim K.H., Kim H. (2011). Potential role of NADPH oxidase-mediated activation of Jak2/Stat3 and mitogen-activated protein kinases and expression of TGF-beta1 in the pathophysiology of acute pancreatitis. Inflamm. Res..

[70] Gerstgrasser A., Melhem H., Leonardi I., Atrott K., Schäfer M., Werner S., Rogler G., Frey-Wagner I. (2017). Cell-specific activation of the Nrf2 antioxidant pathway increases mucosal inflammation in acute but not in chronic colitis. J. Crohn Colitis.

[71] Zagoura D., Canovas-Jorda D., Pistollato F., Bremer-Hoffmann S., Bal-Price A. (2017). Evaluation of the rotenone-induced activation of the Nrf2 pathway in a neuronal model derived from human induced pluripotent stem cells. Neurochem. Int..

[72] Zhang M., Wu Y.-Q., Xie L., Wu J., Xu K., Xiao J., Chen D.-Q. (2018). Isoliquiritigenin protects against pancreatic injury and intestinal dysfunction after severe acute pancreatitis via Nrf2 signaling. Front. Pharmacol..

[73] Rodriguez-Nicolas A., Martinez-Chamorro A., Jimenez P., Matas-Cobos A.M., Redondo-Cerezo E., Ruiz-Cabello F. (2018). TH1 and TH2 Cytokine Profiles as Predictors of Severity in Acute Pancreatitis. Pancreas.

[74] Cao W.L., Xiang X.H., Chen K., Xu W., Xia S.H. (2014). Potential role of NADPH oxidase in pathogenesis of pancreatitis. World J. Gastrointest. Pathophysiol..

[75] Gukovskaya A.S., Gukovsky I., Algul H., Habtezion A. (2017). Autophagy, Inflammation, and Immune Dysfunction in the Pathogenesis of Pancreatitis. Gastroenterology.

[76] Fusco R., Cirmi S., Gugliandolo E., Di Paola R., Cuzzocrea S., Navarra M. (2017). Anti-oxidant and anti-inflammatory effects of a flavonoid-rich extract from orange juice in experimental colitis. Free Radic. Biol. Med..

[77] Leveau P., Wang X., Sun Z., Börjesson A., Andersson E., Andersson R. (2005). Severity of pancreatitis-associated gut barrier dysfunction is reduced following treatment with the PAF inhibitor lexipafant. Biochem. Pharmacol..

[78] Dumnicka P., Maduzia D., Ceranowicz P., Olszanecki R., Drożdż R., Kuśnierz-Cabala B. (2017). The interplay between inflammation, coagulation and endothelial injury in the early phase of acute pancreatitis: Clinical implications. Int. J. Mol. Sci..

[79] Manohar M., Verma A.K., Venkateshaiah S.U., Sanders N.L., Mishra A. (2017). Pathogenic mechanisms of pancreatitis. World J. Gastrointest. Pharmacol. Ther..

