# Supplementary material for: Biochemical Evaluation of the Antioxidant Effects of Hydroxytyrosol on Pancreatitis-Associated Gut Injury
Source: Antioxidants (Basel). 2020 Aug 22;9(9):781. doi: 10.3390/antiox9090781 (PMC7555523; doi:10.3390/antiox9090781)
Supplement: Supplementary file 1 [file antioxidants-09-00781-s001.pdf]

Figure S1: Haematoxylin and Eosin staining of pancreas and colon tissues: pancreas histological analysis: sham + vehicle (A), sham + hydroxytyrosol (HT) (B) caerulein + vehicle (C), caerulein + HT(D), histological injury score (I), colon histological analysis: sham + vehicle (E), sham + HT (F), caerulein + vehicle (G), caerulein + HT(H), histological injury score (J). For the analysis, n=5 animals from each group were employed. A p-value <0.05 was considered significant. * $p < 0.05$ vs. sham, # $p < 0.05$ vs. vehicle, ** $p < 0.01$ vs. sham, ## $p < 0.01$ vs. vehicle, *** $p < 0.001$ vs. sham, ### $p < 0.001$ vs. vehicle.

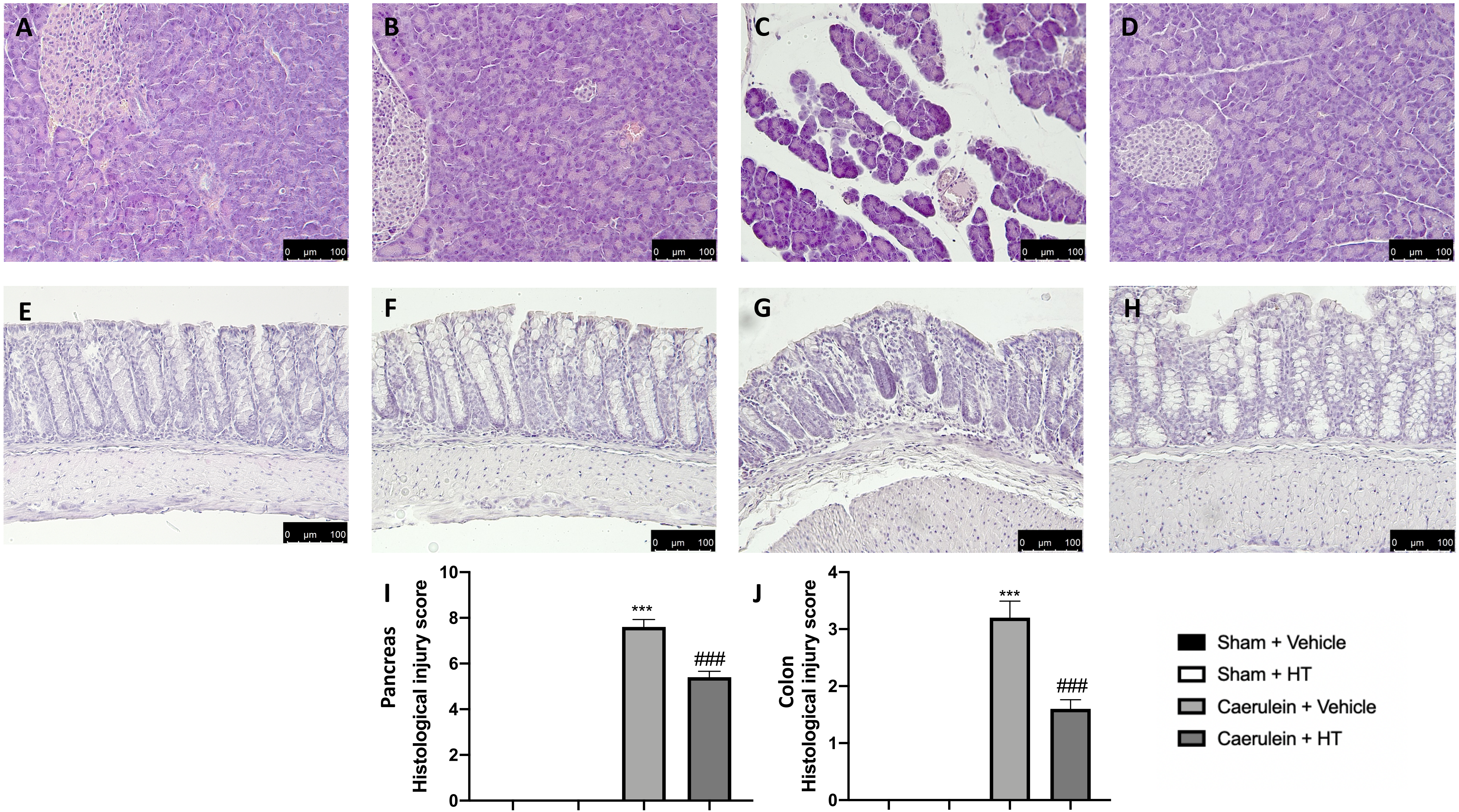
A
B
C
D
E
F
G
H
0 μm 100
I
Pancreas
Histological injury score
***
###
J
Colon
Histological injury score
***
###
Sham + Vehicle
Sham + HT
Caerulein + Vehicle
Caerulein + HT
